# Machine Learning Applications in Mental Health and Substance Use Research Among the LGBTQ2S+ Population: Scoping Review

**DOI:** 10.2196/28962

**Published:** 2021-11-11

**Authors:** Anasua Kundu, Michael Chaiton, Rebecca Billington, Daniel Grace, Rui Fu, Carmen Logie, Bruce Baskerville, Christina Yager, Nicholas Mitsakakis, Robert Schwartz

**Affiliations:** 1 Centre for Addiction and Mental Health Toronto, ON Canada; 2 Dalla Lana School of Public Health University of Toronto Toronto, ON Canada; 3 Factor-Inwentash Faculty of Social Work University of Toronto Toronto, ON Canada; 4 Sunnybrook Research Institute University of Toronto Toronto, ON Canada; 5 Women’s College Research Institute Toronto, ON Canada; 6 Canadian Institutes of Health Research Government of Canada Ottawa, ON Canada; 7 School of Pharmacy, Faculty of Science University of Waterloo Kitchener, ON Canada; 8 Children’s Hospital of Eastern Ontario Research Institute Ottawa, ON Canada

**Keywords:** sexual and gender minorities, mental health, mental disorders, substance-related disorders, machine learning

## Abstract

**Background:**

A high risk of mental health or substance addiction issues among sexual and gender minority populations may have more nuanced characteristics that may not be easily discovered by traditional statistical methods.

**Objective:**

This review aims to identify literature studies that used machine learning (ML) to investigate mental health or substance use concerns among the lesbian, gay, bisexual, transgender, queer or questioning, and two-spirit (LGBTQ2S+) population and direct future research in this field.

**Methods:**

The MEDLINE, Embase, PubMed, CINAHL Plus, PsycINFO, IEEE Xplore, and Summon databases were searched from November to December 2020. We included original studies that used ML to explore mental health or substance use among the LGBTQ2S+ population and excluded studies of genomics and pharmacokinetics. Two independent reviewers reviewed all papers and extracted data on general study findings, model development, and discussion of the study findings.

**Results:**

We included 11 studies in this review, of which 81% (9/11) were on mental health and 18% (2/11) were on substance use concerns. All studies were published within the last 2 years, and most were conducted in the United States. Among mutually nonexclusive population categories, sexual minority men were the most commonly studied subgroup (5/11, 45%), whereas sexual minority women were studied the least (2/11, 18%). Studies were categorized into 3 major domains: web content analysis (6/11, 54%), prediction modeling (4/11, 36%), and imaging studies (1/11, 9%).

**Conclusions:**

ML is a promising tool for capturing and analyzing hidden data on mental health and substance use concerns among the LGBTQ2S+ population. In addition to conducting more research on sexual minority women, different mental health and substance use problems, as well as outcomes and future research should explore newer environments, data sources, and intersections with various social determinants of health.

## Introduction

### Background

Members of the lesbian, gay, bisexual, transgender, queer or questioning, and two-spirit (LGBTQ2S+) population experience significant mental health disparities and are at a higher risk of substance use problems compared with their heterosexual and cisgender peers [1-5]. A meta-analysis of 25 studies revealed that lesbian, gay, and bisexual individuals had 2.47 times increased lifetime risk of attempting suicide, 1.5 times increased risk of depression and anxiety disorders, and 1.5 times increased risk of alcohol and other substance dependence over a 12-month period [2]. Recent statistics from the 2015 National Survey on Drug Use and Health in the United States reported that the sexual minority population have an increased likelihood of past year use of illicit drugs, marijuana, and opioids; current use of cigarettes and alcohol; and past year diagnosis of any mental illness compared with sexual majority groups [6]. Members of the LGBTQ2S+ population also use mental health services and substance use treatment more frequently than cisgender and heterosexual individuals [6,7].

There is a robust evidence base documenting sexual orientation and gender identity as social determinants of health, whereby members of the LGBTQ2S+ population experience stressors from stigma, social, and economic exclusion that contribute to increased mental health challenges and resultant coping strategies, including problematic substance use [8-10]. In addition, intersecting experiences of marginalization such as race, ethnicity, disability, and homelessness; lack of familial and peer support; various acts of bullying, harassment, and hate crimes; and experience of self-stigmatization, such as internalized homophobia, biphobia, and transphobia, contribute to further deterioration of mental health and substance use concerns [8,11-16].

With advances in technology, novel statistical methods, such as machine learning (ML), have emerged as promising means of analyzing a vast range of complex data in public health informatics [17,18]. ML uses computational power to identify or *mine* hidden data patterns and has been increasingly used for content analysis and as a predictive modeling technique [17]. These characteristics are particularly important for investigating mental health and substance use issues among the LGBTQ2S+ population, where social stigma and institutional barriers make sexual and gender identity disclosure difficult, rendering the data invisible [19-21].

There are 3 major types of ML, including (1) supervised learning, (2) unsupervised learning, and (3) semisupervised learning. Supervised learning aims to learn from labeled data to predict the class of unlabeled input data or outcome variables [22]. Unsupervised learning does not require an outcome variable, thereby allowing the algorithm to freely detect and recognize hidden patterns with minimal human interference [22,23]. Semisupervised learning learns from both labeled and unlabeled data, where it can use readily available unlabeled data to improve supervised learning tasks when the labeled data are scarce or expensive [24]. A more advanced form of ML, deep learning, has gained popularity in health research in recent years and uses an artificial neural network model with multiple layers to hierarchically define and process data [25]. These ML methods provide the opportunity to understand data more thoroughly and effectively, as well as yield meaningful predictions beyond traditional statistical methods.

Several reviews, including 3 recent systematic reviews, have been conducted to summarize the application of ML in substance use and mental health issues [23,26-28]. These systematic reviews have reported ML applications in 54 articles on mental health, 87 articles on suicidal behavior, and 17 articles on addiction research and reported good performance in predicting human behavior [23,26,28]. However, most of these reviews and studies focused on broad categories and the general population or patient records.

### Objectives

Although one scoping review has explored studies that predict population-specific health with ML [29], the study did not identify ML applications among the LGBTQ2S+ population. There is a substantial gap in the literature, with no existing review focused on ML studies examining mental health and substance use among the LGBTQ2S+ population. As a result, we conducted a scoping review to address these knowledge gaps with the aim of mapping the current status of ML studies, focusing on this field and identifying the research gap to facilitate future research. Regarding persistent mental health and problematic substance use concerns and disparities among the LGBTQ2S+ population, the findings from this review will provide useful insights to inform research and programs.

## Methods

### Objectives and Methodology Framework

This review aims to conduct a comprehensive search of studies using ML to investigate mental health or substance use among LGBTQ2S+ communities and to determine the scope of future research. We used the following 5-stage methodological framework developed by Arksey and O’Malley [30]: (1) identifying specific research questions; (2) identifying relevant studies through a comprehensive search of different sources; (3) study selection by applying inclusion and exclusion criteria; (4) data charting using custom-made data extraction forms; and (5) collating, summarizing, and reporting the results. We also used an extension of the Preferred Reporting Items for Systematic Reviews and Meta-Analyses guidelines for scoping reviews [31] to present our findings, and the Joana Briggs Institute proposed methodology of scoping reviews [32] to narrate the implications for future research. The review protocol was registered on the Open Science Framework [33] on December 17, 2020, to facilitate transparency and reproducibility of the study.

### Identifying Research Questions

Initially, we identified a broad set of preliminary questions for this scoping review:

- What is the volume of the literature that used machine learning analysis in the field of mental health and substance use among the LGBTQ2S+ population?
- What are the fields of mental health and substance use among the LGBTQ2S+ population that have been studied by machine learning?
- Which subgroups of the LGBTQ2S+ population have been investigated? Are there any specific subgroups that have been studied using machine learning analysis?
- What types of machine learning methods (eg, supervised, unsupervised, semisupervised, and deep learning) and algorithms (eg, decision trees, random forest, logistic regression, and penalized regression) have been used to study LGBTQ2S+ mental health and substance use?
- What are the real-world implications of these studies? Are there any knowledge gaps or untouched domains that should be addressed in future research?

### Identifying Relevant Studies

To gather a large quantity of relevant literature, we followed previous review studies with similar objectives [27,29] and searched the following databases: MEDLINE (Ovid), Embase (Ovid), CINAHL Plus, APA PsycINFO (Ovid), PubMed, and IEEE Xplore. We also searched the Summon (ProQuest) database used by the University of Toronto Libraries, which searches across many other databases, journal packages, e-book collections, and other resources. Information technology databases such as IEEE Xplore were selected as a potential source of ML-related literature. Literature searches involved a combination of keywords (eg, *mental health, mental disease, mental health service, substance abuse, ML, sexual and gender minorities, LGBT, lesbian, gay, men who have sex with men, bisexual, queer, two-spirit, intersex, and transgender*) and medical subject headings, if applicable. A librarian was consulted regarding the keywords and search terms.

Two reviewers (AK and RB) conducted the database search from November 25 to December 13, 2020, and imported all citations to the Covidence web platform, where duplicate papers were removed automatically. The databases were searched from the date of inception of the databases to the year 2020, with no filter in place for publication year. The bibliography lists of the included studies and review papers were reviewed on December 13, 2020, to identify any potential studies. The full Embase search strategy, representing an example of the search query applied to all other databases, is presented in Multimedia Appendix 1.

### Study Selection

We included studies that used ML to investigate mental health or substance use behaviors of people within the LGBTQ2S+ population. Studies in which ML was used partially, but not for the main statistical analysis, were included in the review. We only included empirical investigations, thereby excluding editorials, opinion pieces, and reviews. We also excluded papers that used logistic regression analyses, not as a ML algorithm, but to determine LGBTQ2S+ identity status. In addition, studies in which full texts could not be retrieved with institutional license, and studies of genomics, pharmacokinetics, and those that were not directly relevant to humans were excluded.

Two reviewers (AK and RB) independently screened each title and abstract based on the eligibility criteria and completed full-text screening of the remaining studies. Disagreements were resolved through discussions among the 3 reviewers (AK, RB, and MC) to yield a list of final included studies.

### Data Charting

To facilitate data charting and reporting, individual reviewers (AK and RB) first reviewed all studies and extracted key phrases and concepts from each study. We based our data extraction items on features identified in a recent biomedical guideline for reporting ML studies [34]. Custom-made data extraction forms were developed from this guideline, which included major extraction categories such as general study characteristics (ie, author, year, country, target population, source of data, sample size, field of study, ML domains, ML methods, algorithms, and outcomes), key components of model development (ie, whether the studies discussed methods of feature selection, resampling, model performance metrics, and method of validation), and discussion of study findings (ie, importance ranking of features, intersectionality, and other procedures or features applied).

### Collating, Summarizing and Reporting Results

We presented descriptive statistics for the extracted data sets by calculating the total number and percentage of all studies in each category. To provide a visual overview of the range of data, we presented a bar chart that showed the frequency analysis of studies according to the field of study and a pie chart that demonstrated the proportion of studies in the major domains of ML. We used a narrative synthesis approach [35] to describe the findings of the studies in the different ML domains and explored relationships in the data. Finally, we discussed research gaps to facilitate future research.

## Results

**Figure 1:**
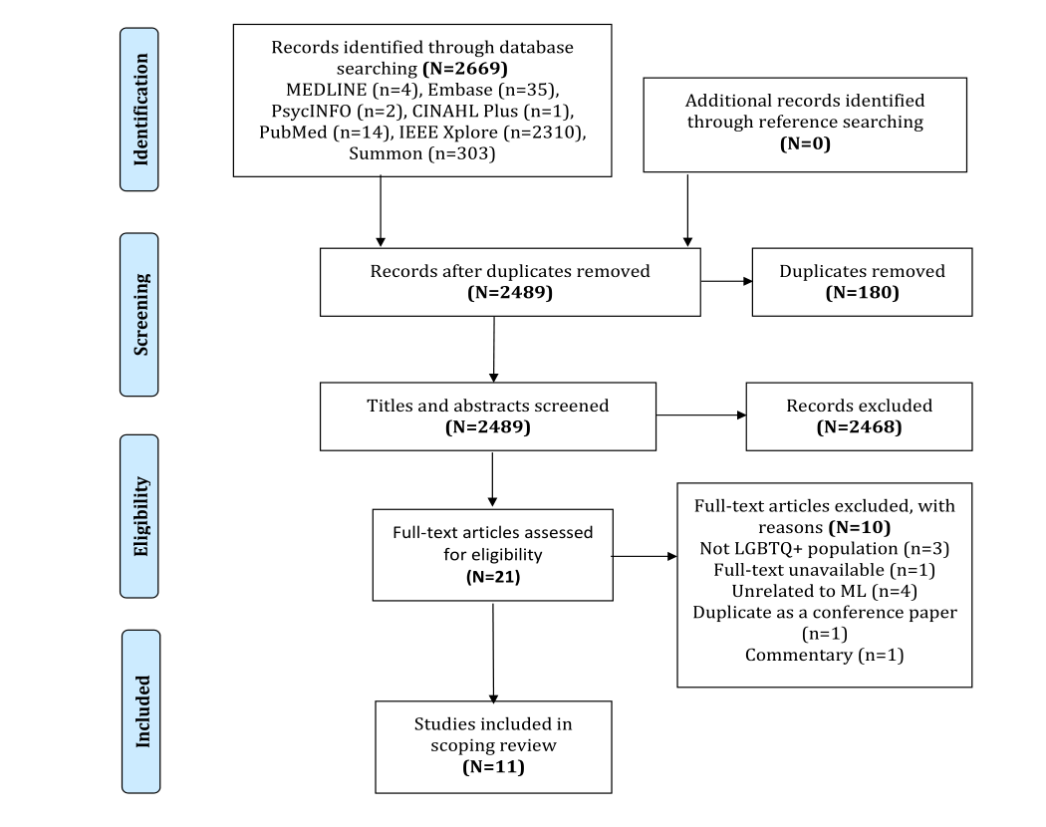
Preferred Reporting Items for Systematic Reviews and Meta-Analyses flow diagram documenting study exclusion. LGBTQ+: lesbian, gay, bisexual, transgender, queer, or questioning; ML: machine learning.

The initial search of databases yielded 2669 articles, of which 2489 were retrieved after removing duplicates. We also searched the reference lists of potentially eligible articles and previous reviews but could not identify any studies that matched our inclusion criteria. After title and abstract screening, 21 articles were selected for full-text screening. Of these, we excluded articles that did not meet the target population criteria of the LGBTQ2S+ population (3/21, 14%), full-texts could not be retrieved (1/21, 4%), unrelated to ML (4/21, 19%), duplicate article published in a conference proceeding (1/21, 4%), and a commentary (1/21, 4%). This resulted in 11 studies being included in the final review [36-46]. The detailed selection process of the articles is presented in the Preferred Reporting Items for Systematic Reviews and Meta-Analyses (PRISMA) flow diagram (Figure 1).

### Study Characteristics

All 11 included studies [36-46] were published within the last 2 years (Table 1). Most of the studies were carried out in the United States (7/11, 63%) [36,38,39,41-43,45]. Among the target population categories that were not mutually exclusive, sexual minority men (gay, men who have sex with men, bisexual) were the most commonly studied (5/11, 45%) subgroups [37,40,42-44], followed by transgender (3/11, 27%) [39,45,46] and LGBTQ+ (3/11, 27%) [36,38,41] people at large, whereas sexual minority women (lesbian and bisexual) (2/11, 18%) [43,45] were the least commonly represented populations. None of the studies included two-spirit persons as their target population (Table 1).

**Table 1:** Summary statistics of included studies (N=11) [36-46].^a^

Characteristics				Number of studies, n (%)
**Countries**				
	United States			7 (63)
	China			2 (18)
	Sweden			1 (9)
	Australia			1 (9)
**Years published**				
	2019			5 (45)
	2020			6 (54)
**Field of study**				
	**Mental health (n=9)**			
		Suicide or self-injury	2 (18)	
		Depression	2 (18)	
		Mood or affect processes	3 (27)	
		Minority stress	1 (9)	
		Gender incongruence	1 (9)	
	**Substance use (n=2)**			
		Tobacco	1 (9)	
		Poppers or alkyl nitrites	1 (9)	
**Target population^b^**				
	Sexual minorities: male (gay, MSM^c^, bisexual)			5 (45)
	Sexual minorities: female (lesbian, bisexual)			2 (18)
	Transgender or gender minorities			3 (27)
	LGBT/LGBTQ+^d^			3 (27)
**Domains of ML^e^**				
	Web content analysis			6 (55)
	Prediction modeling			4 (36)
	Imaging study			1 (9)
**Type of ML**				
	Supervised			9 (82)
	Unsupervised			3 (27)
	Deep			1 (9)
**ML algorithms**				
	LDA^f^			3 (27)
	RF^g^			2 (18)
	SVM^h^			2 (18)
	CNN^i^			1 (9)
	MLP^j^			1 (9)
	NB^k^			1 (9)
	Penalized regression (LASSO^l^, elastic net regularized regression, ridge regression)			2 (18)
	Logistic regression			1 (9)
	Boosting (XGBoost^m^, AdaBoost^n^, GBM^o^)			3 (27)
	Classification tree			2 (18)
**Feature selection**				
	Yes			7 (64)
	No			4 (36)
**Discussed model performance**				
	Used performance metrics			9 (82)
	Didn't use performance metrics			1 (9)
	Didn't discuss performance			1 (9)
**Method of validation**				
	Hold-out			2 (18)
	Cross-validation			7 (64)
	External validation			2 (18)
	Unspecified			4 (36)

^a^Multiple response options were possible for some study characteristics.

^b^Categories are not mutually exclusive.

^c^MSM: men who have sex with men.

^d^LGBT/LGBTQ+: lesbian, gay, bisexual, and transgender/lesbian, gay, bisexual, transgender, queer, or questioning.

^e^ML: machine learning.

^f^LDA: latent Dirichlet allocation.

^g^RF: random forest.

^h^SVM: support vector machine.

^i^CNN: convolutional neural network.

^j^MLP: multilayered perceptron.

^k^NB: Naive Bayes.

^l^LASSO: least absolute shrinkage and selection operator.

^m^XGBoost: eXtreme Gradient Boosting.

^n^AdaBoost: Adaptive Boosting.

^o^GBM: Generalized Boosted Model.

Most of the studies focused on mental health (9/11, 82%) [36-42,45,46], and only 18% (2/11) studies [43,44] focused on substance use concerns. Most studies examined several mental health issues, such as depression, suicide, mood or affect processes, minority stress, and gender incongruence [36-42,45,46], whereas other studies that focused on substance use only examined tobacco and poppers or alkyl nitrites use [43,44]. No study looked into mental health issues and substance use concerns among the LGBTQ2S+ population simultaneously (Table 1).

The studies were categorized into 3 major ML domains: web content analysis, prediction modeling, and imaging study. Over half of the studies (6/11, 55%) were identified as web content analysis [36-41], and 36% (4/11) were identified as prediction modeling [42-45]; 1 study (9%) was identified as an imaging study [46] (Table 1).

The most commonly used class of ML methods was supervised (9/11, 82%) [37-39,41-46], followed by unsupervised (3/11, 27%) [36,37,40] and deep learning (1/11, 9%; Table 1) [41]. The most frequently used ML algorithms were latent Dirichlet allocation (3/11, 27%) and boosting (3/11, 27%), followed by random forest, support vector machines, penalized regression (ie, least absolute shrinkage and selection operator, elastic net regularized regression, and ridge regression), classification tree, logistic regression, naive Bayes, multilayered perceptron, and convolutional neural network (Table 1).

Approximately two-thirds (7/11, 64%) of the studies [37,38,42-46] discussed their methods of feature selection, among which the median number of features used was 19. Most of the studies used cross-validation methods (7/11, 64%) [37-39,41,44-46], especially 10-fold cross-validation. Furthermore, 18% (2/11) of the articles used the hold out method [39,41], 18% (2/11) used external validation [37,41], and 36% (4/11) articles [36,40,42,43] did not report how they validated their method. Most studies (9/11, 82%) [36-39,41-43,45,46] used at least one performance metric (eg, area under ROC curve, precision-recall, or F1 score) to discuss model performance. However, the remaining studies either did not use any performance metric [44] or did not discuss any model performance [40] (Table 1).

### Machine Learning Domains

Multimedia Appendix 2 summarizes the characteristics of the final 11 included studies [36-46] and Multimedia Appendix 3 [36-46] presents the ML methodology used in the studies.

The 54% (6/11) studies [36-41] in the web content analysis domain obtained their data from social media sources such as Twitter, Blued, Tumblr, Reddit, and LGBT Chat and Forums. The volume of data used ranged from 12,000 to 41 million web posts. Half of the studies used their data to analyze the mood or affect processes of the users related to their sexual and gender identities [39-41] (Multimedia Appendix 2).

Among the 4 studies in the prediction modeling domain, 50% (2/4) of the studies analyzed data on adult participants [42,44] and 50% (2/4) on adolescents [43,45]. Only 1 study used a public health data set of 28,811 participants [43]; other studies used either cross-sectional or cohort data from longitudinal studies [42,44,45]. Half of the studies focused on mental health (depression and suicide) [42,45] and half on substance use behavior (cigarette, e-cigarette, and poppers use) [43,44] (Multimedia Appendix 2). Of the 4 studies, only 25% (1/4) study [45] ranked their feature importance, and 50% (2/4) studies [42,45] examined intersectionalities (Multimedia Appendix 3). One study investigated the intersection of income and other social and environmental stressors with racial or ethnic disparities and its impact on depressive symptomology among men who have sex with men [42], whereas the other focused on the intersection between various social and behavioral determinants of health (self-image, race, education, socioeconomic status, family support, friends, stigma, discrimination, etc) as risk factors of self-injurious behaviors among sexual and gender minority women [45].

One imaging trial study used clinical and functional magnetic resonance imaging data of 25 transgender adults to identify the relationship between pretherapy functional brain connectivity and posthormone therapy body congruence [46]. All 4 studies [42-45] of the prediction modeling domain and 1 imaging study [46] used the supervised method of ML, whereas studies in the web content analysis domain [36-41] used supervised (4/11, 36%), unsupervised (3/11, 27%), and deep learning (1/11, 9%) methods (Multimedia Appendix 3).

## Discussion

### Principal Findings

Our results show that the application of ML to assess mental health and substance use behavior among the LGBTQ2S+ population is still new in health research, compared with the increasing use of ML techniques in other health research domains. Although there is continued criminalization and lack of LGBTQ2S+ rights protection in 67 United Nations member states at the end of 2020 [47], there appears to be an increasing acceptance of sexual and gender minority people in diverse contexts such as in North American countries and Western Europe [48]. However, very few of the included studies were conducted outside the United States (Table 1).

Only a few mental health problems were addressed across the few relevant ML studies conducted to date (Table 1). Although there is evidence of a higher prevalence of anxiety disorders, posttraumatic stress disorder, and various mood disorders (eg, mania and persistent depressive disorder) among the LGBTQ2S+ population compared with cisgender and heterosexual counterparts [4], no studies have been conducted on these issues. Compared with mental health issues, substance use problems among the LGBTQ2S+ population were almost untouched. Moreover, both of the included substance use related studies predicted the present use of substances [43,44], and no studies have examined future substance use, cessation, or substance use treatment-seeking behavior.

Underlying factors behind the low number of ML studies on mental health and substance use issues among the LGBTQ2S+ population may be sex and gender identity-related data invisibility and social and institutional bias [21,49]. Electronic health records have been used as a common and promising data source for ML techniques to predict population health in other research areas [27,29]. However, binary representation of sex and gender (ie, man or woman) in the electronic health records system makes some data unavailable for analysis by ML, which can underrepresent the actual problem [21,50,51]. Adopting inclusive gender, sex, and sexual orientation (GSSO) information practices, collecting sexual and gender diversity, has the potential to ensure data justice, alleviate unintentional bias, and reduce health inequity [49]. A good example of inclusive GSSO information practice could be the proposed equity stratifiers by the Canadian Institute of Health Information [52]. However, other potential data sources of ML applications, such as social media, cross-sectional survey data, longitudinal cohort, and administrative data sets were used in the included studies (Multimedia Appendix 2).

Most studies were in the web content analysis domain, indicating social media to be a potentially useful epidemiological resource for collecting data on LGBTQ2S+ people and analyzing the data using ML (Multimedia Appendix 2). We observed that unsupervised ML has also been applied in these studies with data drawn from social media [36,37,40], thus holding the potential to support qualitative research by handling large textual data sets with its computational power. This is particularly useful in LGBTQ2S+ health research, given the stigma-related and structural barriers toward identity disclosure that may inhibit data collection through other methodologies [50,51,53,54]. The use of ML in these studies has shown potential for automated identification of at-risk individuals for crisis suicide prevention and intervention [36], depressive emotions [37], minority stressors [38], negative emotions [40], and mental health signals [41] among the LGBTQ2S+ community. In addition, the sequence of transgender identity disclosure identified in a study by Haimson et al [39] may guide resource allocation and provide support through gender transition. However, self-reported mental health problems on social media might not reflect clinical diagnoses or symptomologies.

Although there is evidence of the influence of intersections of various social and behavioral determinants of health on the increased prevalence of mental health and substance use concerns among the LGBTQ2S+ population [11-16], only 2 studies examined the intersection of sexual and gender identity with ethno-racial identities, and several social, economic, and behavioral factors (ie, income, social stigma, discrimination, and family support), and their impact on depression and self-injurious behaviors [42,45]. No such studies in our review explored intersectionality in the field of substance use. Identifying these intersections by leveraging ML techniques would have practical implications by determining risk and protective factors as well as informing strategies for promoting mental well-being and substance use prevention and intervention with and for LGBTQ2S+ people. In the context of various techniques used in intersectional research, both qualitative and quantitative, and recent trends in mixed methods research [55], ML can be a very useful tool for processing vast quantities of data, data mining and clustering, and classifying attribute relationships [56,57]. Apart from the partial dependency-based measures, newer techniques and methods [58,59] in ML have emerged for analyzing interaction effects and are more suitable for assessing intersectionality.

Following the current guidelines for reporting ML studies in biomedical research [34], we documented a range of explanatory findings seen in the included studies and found that most studies mentioned their performance metrics, method of feature selection, and method of validation of their model (Table 1 and Multimedia Appendix 3). However, only 27% (3/11) studies [37,38,45] adopted the approach of approximating a relative importance score of individual features that reflected their overall contributions to the model (Multimedia Appendix 3). The implications of providing an importance score to features are particularly valuable for predictive modeling studies, where the most important predictors are targeted for future strategy adoption. Another notable finding was about half (n=2) [42,43] of the predictive modeling studies did not report any method of validation, and none of them conducted external validation of the resulting model on a different population (Multimedia Appendix 3). Validation is an important aspect of the predictive modeling process, which increases the reproducibility and generalizability of the model [60]. Hence, future studies in this domain should follow existing guidelines to validate their models [34]. Moreover, half of the predictive modeling studies had small sample sizes (<1000) (Multimedia Appendix 2). Small data sets can affect the model performance [61]. Using large population-based data sets for future research can overcome this problem and fully leverage the benefits of ML.

Compared with the other 2 domains, there was a significant gap in ML research using imaging data (ie, functional magnetic resonance imaging or electroencephalography) to examine mental health and substance use among the LGBTQ2S+ population (Table 1). Although a single identified imaging study [46] predicted cross-sex hormonal therapy responsiveness in the transgender population, which is useful for guiding and selecting candidates for therapy, the sample size was small, limiting the generalizability of the findings.

### Future Research Directions

We detected significant research gaps in ML applications for mental health and substance use research among the LGBTQ2S+ population. First, future research should investigate other mental health issues (ie, anxiety disorders and mood disorders) and substance use behavior and problems (ie, alcohol, opioids, and illicit drugs) among the LGBTQ2S+ population. Second, the potential of ML applications in predicting substance use related outcomes (ie, cessation, overdose events, routes of administration, driving impairments, and other adverse reactions), mental health service access, and mental health-related outcomes (ie, disabilities, symptom management, suicide and suicide attempts, economic burden, and health care costs) should be explored.

Third, further research is needed on sexual minority women. The small number of studies included (Table 1) did not allow exploration of shared and different health needs and priorities between and within the LGBTQ2S+ population. Fourth, as the legal and societal context in which the LGBTQ2S+ population lives differ significantly between countries [48], more research should be conducted in countries outside the United States. Fifth, specific research initiatives targeted at investigating the intersection of sexual and gender minority identity with other social determinants of health (ie, race, ethnicity, citizenship, socioeconomic status, and housing condition) are necessary to better understand their potential for fostering risk and resilience regarding mental health and substance use. Finally, different data sources should be used in ML studies. Large-population-level administrative data sets should be used for prediction modeling studies for the accurate application of ML models. In addition, with the advancement of technology, the digitalization of health care, and where LGBTQ2S+ status is captured in electronic health records, these health records can be a potential data resource for ML studies with real-world clinical implications for LGBTQ2S+ people.

### Strength and Limitations

To the best of our knowledge, our review is the first of its kind to explore the use of ML applications in examining mental health and substance use among LGBTQ2S+ populations. We adopted a comprehensive search strategy, including searching various multidisciplinary peer-reviewed databases to identify relevant articles as much as possible. The findings of our review need to be interpreted with consideration of one key limitation. Owing to the small number of studies, highly heterogeneous characteristics of the included studies, and inconsistent reporting of model development and validation, we could not perform a critical appraisal of the studies and therefore could not comment significantly on the overall performance of the ML techniques. However, we followed the approaches of previous scoping reviews with similar objectives [27,29] and were interested in understanding the general topics or areas being investigated by ML in the field of mental health and substance use among the LGBTQ2S+ population (ie, most commonly used data sources, study countries, and study populations) and identifying research gaps to inform future research.

As more studies are published on this research topic in the future, a systematic review with critical appraisal of relevant literatures should be conducted as the next step in research. Researchers are attempting to expand established reporting guidelines to include items that accommodate ML studies, such as the Transparent Reporting of a Multivariable Prediction Model for Individual Prognosis or Diagnosis statement specific for M [62], the Artificial Intelligence extension for Consolidated Standards of Reporting Trials [63], and Artificial Intelligence extension for Standard Protocol Items: Recommendations for Interventional Trials [63] guidelines. Once developed, these guidelines can be used as critical appraisal tools for studies that adopt ML-based data analysis. There is also an opportunity to incorporate fairness and equity considerations in the development of appraisal tools for ML studies. Preliminary research has already developed mathematical metrics to measure the fairness of a ML algorithm, and if intersectionalities are met in the models [64].

### Conclusions

Although there is an exponential growth of ML applications in other health research sectors, few studies have used these techniques in the field of mental health and substance use among the LGBTQ2S+ population. In addition to undertaking more research, future researchers should focus on applying ML algorithms with considerations for real-world implications through public health interventions and adopting policies that aim to improve health equity.

## Appendix

Multimedia Appendix 1 Embase search query.

Multimedia Appendix 2 Summary of studies using machine learning analysis in mental health and substance use among lesbian, gay, bisexual, transgender, queer or questioning, and two-spirit population (N=11).

Multimedia Appendix 3 Summary of characteristics of machine learning methods used (N=11).

## Abbreviations

LGBTQ2S+: lesbian, gay, bisexual, transgender, queer or questioning, and two-spirit
ML: machine learning
PRISMA: Preferred Reporting Items for Systematic Reviews and Meta-Analyses
