# Supplementary material for: Machine Learning Applications in Mental Health and Substance Use Research Among the LGBTQ2S+ Population: Scoping Review
Source: JMIR Med Inform. 2021 Nov 11;9(11):e28962. doi: 10.2196/28962 (PMC8663464; doi:10.2196/28962)
Supplement: Multimedia Appendix 1 [file medinform_v9i11e28962_app1.docx]

Embase search query.

| **Search Terms** |
| --- |
| 1. exp mental health/ |
| 2. exp mental disease/ |
| 3. exp mental health care/ |
| 4. exp mental health service/ |
| 5. ((mental or psychiatric or depressi* or anxiety or mood or bipolar or eating or schizophren* or Psycho* or suicid*) adj2 (disorder* or issue* or condition* or ideation or attempt*)).tw,kw. |
| 6. exp substance abuse/ |
| 7. exp drug dependence/ |
| 8. exp drug dependence treatment/ |
| 9. exp harm reduction/ |
| 10. ((Substance or drug or Alcohol or cannabis or Marijuana or cocaine or opioid* or tobacco or nicotin*) adj2 (use* or abuse* or dependen* or addicti* or withdraw* or cessation or treat*)).tw,kw. |
| 11. 1 or 2 or 3 or 4 or 5 or 6 or 7 or 8 or 9 or 10 |
| 12. exp machine learning/ |
| 13. ((supervised or unsupervised or deep or machine) adj2 learning).tw,kw. |
| 14. 12 or 13 |
| 15. exp LGBT people/ |
| 16. (Lesbian or gay or bisexual* or Homo or homosexual* or MSM or men sex with men or queer or two-spirit or transgender or intersex or LGBT*).tw,kw. |
| 17. 15 or16 |
| 18. 11 and 14 and 17 |
