# Supplementary material for: Machine Learning Applications in Mental Health and Substance Use Research Among the LGBTQ2S+ Population: Scoping Review
Source: JMIR Med Inform. 2021 Nov 11;9(11):e28962. doi: 10.2196/28962 (PMC8663464; doi:10.2196/28962)
Supplement: Multimedia Appendix 2 [file medinform_v9i11e28962_app2.docx]

Summary of studies using ML analysis in mental health and substance use among LGBTQ2S+^a^ population (N=11).

| Author(s) and year | | Target population | Sample size | Data input | Source of data | Field of study | Outcome(s) |
| --- | --- | --- | --- | --- | --- | --- | --- |
| **Web content analysis** | | | | | | | |
|  | Liang et al, 2019 [36] | LGBT^b^ | 65,000 | Posts on social media | LGBT Chat and Forums | Suicide | Help-seeking behavior related topics |
|  | Li et al., 2020 [37] | MSM^c^ and non-MSM | 41 million | Posts on social media | Blued and Twitter | Depression | Depression emotion |
|  | Saha et al, 2019 [38] | LGBTQ+^d^ | 12,000 | Posts on social media | Reddit | Minority stress | Prejudice events, perceived stigma, internalized stigma |
|  | Haimson et al, 2020 [39] | Transgender | 41,000 | Posts on social media | Tumblr | Emotional response to sexual identity disclosure | Self-reported identity disclosure posts |
|  | Huang et al., 2019 [40] | Gay men | 1.6 million (posts), 5 million (votes) and 407,000 (comments) | Posts, votes and comments on social media | Blued | Mood/affect processes | Positive and negative emotions related to sensitive topics, voting outcome in 7 categories including drug use |
|  | Zhao et al, 2020 [41] | LGBTQ+ | 2.3 million | Posts on social media | Twitter | Mood/affect processes | Expressing positive emotions, negative emotions, anger, anxiety, sadness |
| **Prediction modelling** | | | | | | | |
|  | Barrett et al, 2020 [42] | MSM | 1729 | Clinical data | Multicenter AIDS cohort study | Depression | Clinically significant depressive symptoms (CES-D^e^ score≥20) |
|  | Azagba et al., 2019 [43] | Heterosexual and LGB | 28,811 | Person-level survey data | YRBSS^f^ 2015 and 2017 | Cigarette smoking and e-cigarette use | Self-reported cigarette smoking status in past 30 d |
|  | Demant et al, 2019 [44] | Sexual minority men (gay, bisexual, other) | 836 | Person-level survey data | Cross-sectional study | Poppers (alkyl nitrites) use | Self-reported poppers use in past 3 mo |
|  | Smith et al, 2020 [45] | Lesbian, bisexual and questioning females, gender minorities | 252 | Person-level survey data | Longitudinal cohort study | Suicide/self-injury | Self-reported self-injurious thoughts and behaviors in past 6 mo follow-up period |
| **Imaging study** | | | | | | | |
|  | Moody et al, 2020 [46] | Transgender | 25 | Clinical and imaging data | Clinical cohort | Gender incongruence | Body index score, fMRI^g^ images |

^a^LGBTQ2S+: lesbian, gay, bisexual, transgender, queer or questioning, and two-spirit.

^b^LGBT: lesbian, gay, bisexual and transgender.

^c^MSM: men who have sex with men.

^d^LGBTQ+: lesbian, gay, bisexual, transgender, queer or questioning.

^e^CES-D score: Center for Epidemiologic Studies Depression Scale score.

^f^YRBSS: Youth Risk Behavior Surveillance System.

^g^fMRI: functional magnetic resonance imaging.
