# Supplementary material for: Machine Learning Applications in Mental Health and Substance Use Research Among the LGBTQ2S+ Population: Scoping Review
Source: JMIR Med Inform. 2021 Nov 11;9(11):e28962. doi: 10.2196/28962 (PMC8663464; doi:10.2196/28962)
Supplement: Multimedia Appendix 3 [file medinform_v9i11e28962_app3.docx]

Summary of characteristics of ML^a^ methods used (N=11).

| Author(s) and year | | Type of ML | ML algorithm(s) | Feature selection | Re-sampling | Model performance | Method of validation | Importance ranking | Intersectionality | Features/data items |
| --- | --- | --- | --- | --- | --- | --- | --- | --- | --- | --- |
| **Web content analysis** | | | | | | | | | | |
|  | Liang et al, 2019 [36] | Unsupervised | LDA^b^ | —^c^ | — | ✓^d^ | NS^e^ | — | — | Free texts |
|  | Li et al., 2020 [37] | Supervised; Unsupervised | XGBoost^f^; LDA | ✓ | ✓ | ✓ | Stratified 5-fold cross validation; external validation | ✓ | — | 19 features (eg, user profile, social interaction, emotion, linguistic) |
|  | Saha et al, 2019 [38] | Supervised | NB^g^; logistic regression; RF^h^; SVM^i^; MLP^j^ | ✓ | ✓ | ✓ | Stratified κ-fold cross-validation (*k*=5) | ✓ | — | 659 language features expressing sentiment, stigma and symptomatic mental health concerns |
|  | Haimson et al, 2020 [39] | Supervised | Adaptive boosting | — | ✓ | ✓ | 10-Fold cross validation; Hold out | — | — | Free texts |
|  | Huang et al., 2019 [40] | Unsupervised | Twitter-LDA | — | — | — | NS | NA^k^ | — | Free texts |
|  | Zhao et al, 2020 [41] | Supervised; Deep learning | RF; SVM; CNN^l^ | — | ✓ | ✓ | 10-Fold cross validation; Hold out; external validation | NA | — | Free texts |
| **Prediction modeling** | | | | | | | | | | |
|  | Barrett et al, 2020 [42] | Supervised | Classification tree | ✓ | ✓ | ✓ | NS | — | ✓ | Demographics, social, treatment and health exposure |
|  | Azagba et al., 2019 [43] | Supervised | GBM^m^ | ✓ | — | ✓ | NS | — | — | 6 features (eg, age, sex, race, alcohol, cannabis use and attempting suicide) |
|  | Demant et al, 2019 [44] | Supervised | Classification tree (CHAID^n^) | ✓ | ✓ | ✓ | 10-Fold cross validation | — | — | Demographics, poppers and other substance use, LGBT^o^ community-specific, mental and psychosocial well-being |
|  | Smith et al, 2020 [45] | Supervised | LASSO^p^ and elastic net regularized logistic regression | ✓ | ✓ | ✓ | 10-Fold cross validation | ✓ | ✓ | 24 features (eg, demographics, self-injury and suicidal behavior, school, family and social exposure) |
| **Imaging study** | | | | | | | | | | |
|  | Moody et al, 2020 [46] | Supervised | LASSO; ridge regression | ✓ | ✓ | ✓ | N-5 cross-validation | — | — | 10 features (eg, demographics, Kinsey scores, body index scores, BMI therapy duration) |

^a^ML: machine learning.

^b^LDA: latent Dirichlet allocation.

^c^—: Absent

^d^✓ : Present

^e^NS: not specified.

^f^XGBoost: eXtreme Gradient Boosting.

^g^NB: Naïve Bayes.

^h^RF: random forest.

^i^SVM: support vector machine.

^j^MLP: multilayered perceptron.

^k^NA: not applicable.

^l^CNN: convolutional neural network.

^m^GBM: Generalized Boosted Model.

^n^CHAID: χ^2^ Automatic Interaction Detection.

^o^LGBT: lesbian, gay, bisexual and transgender.

^p^LASSO: least absolute shrinkage and selection operator.
